# Intratumoral Susceptibility Signals Reflect Biomarker Status in Gliomas

**DOI:** 10.1038/s41598-019-53629-w

**Published:** 2019-11-19

**Authors:** Ling-Wei Kong, Jin Chen, Heng Zhao, Kun Yao, Sheng-Yu Fang, Zheng Wang, Yin-Yan Wang, Shou-Wei Li

**Affiliations:** 1 Department of Neurosurgery, Sanbo Brain Hospital, Capital Medical University, Beijing, China; 2 Department of Neurosurgery, Yantaishan Hospital, Yantai, China; 3 Department of Pathology, Sanbo Brain Hospital, Capital Medical University, Beijing, China; 4 Beijing Neurosurgical Institute, Capital Medical University, Beijing, China; 5 Department of Neurosurgery, Beijing Tiantan Hospital, Capital Medical University, Beijing, China

**Keywords:** Surgical oncology, Predictive markers, Cancer imaging

## Abstract

Susceptibility-weighted imaging (SWI) can be a useful tool to depict vascular structures in brain tumors as well as micro-bleedings, which represent tumor invasion to blood vessels and could also be representative of tumoral angiogenesis. In this study, we investigated the relationship between SWI features and glioma grades, and the expression of key molecular markers isocitrate dehydrogenase 1 (*IDH1*), O-6-methylguanine-DNA methyltransferase (*MGMT*), and 1p19q. The gliomas were graded according to the intratumoral susceptibility signals (ITSS). We used the Mann-Whitney test to analyze the relationship between ITSS grades and the pathological level and status of these markers. Additionally, the area under the curve (AUC) was used to determine the predictive value of glioma SWI characteristics for the molecular marker status. In these cases, the ITSS grades of low-grade gliomas (LGG) were significantly lower than those of high-grade gliomas (HGG). Similarly, the ITSS grades of gliomas with *IDH1* mutations and *MGMT* methylation were significantly lower than those of gliomas with Wild-type *IDH1* and unmethylated *MGMT*. However, ITSS grades showed no relationship with 1p19q deletion status, while they did show significant predictive ability for glioma grade, *IDH1* mutation, and *MGMT* methylation. These findings indicate an association between some molecular markers and cerebral microbleeds in gliomas, providing a new avenue for non-invasive prediction of molecular genetics in gliomas and an important basis for preoperative personalized surgical treatment based on molecular pathology.

## Introduction

Gliomas, which represent the most common type of primary malignant brain tumor, have an average annual incidence of 3–8 per 100,000 population and a survival period of about 10–20 months^1^. According to the 2016 World Health Organization (WHO) Classification of Tumors of the Central Nervous System^2^, gliomas are further categorized on the basis of both histopathologic features and molecular characteristics, mainly because the importance of molecular types in tracing tumor origin and patient prognosis is being gradually recognized^3^. Among the numerous molecular characteristics of glioma, *IDH* gene mutations are the most critical, of which *IDH1* mutation is closely associated with a good prognosis and favorable survival in patients^4^. Furthermore, patients with gliomas carrying the combined 1p19q deletion have a relatively favorable survival prognosis^5^, while *MGMT* methylation status is an important predictor of the glioma’s sensitivity to alkylating agent chemotherapy^6,7^. However, assessments of these molecular markers are usually performed during pathological examinations after the resection of the tumor, and the molecular characteristics of the tumor are not known before surgery. Thus, the availability of non-invasive methods reflecting these molecular markers associated with the degree of malignancy of the tumor can facilitate individualized surgical treatment.

Susceptibility-weighted imaging (SWI)^8,9^ is a magnetic resonance imaging (MRI) sequence that is sensitive to paramagnetic substances such as hemosiderin deposition and mineral deposits. SWI exploits the susceptibility differences among tissues and uses the phase image to detect these differences. This image is combined with the magnitude image to create an enhanced contrast magnitude image, which is commonly used for detection of neovascularity (venous blood), microhemorrhage, and calcification inside the glioma^10^. Thus, the enhanced sensitivity of SWI for gliomas yields a better contrast in the identification of tumor boundaries and tumor hemorrhage, thereby enabling the determination of the tumor status, including the location of microhemorrhages and the degree of hemorrhage in gliomas. Moreover, the extent of microhemorrhage in different gliomas may be related to heterogeneity in their biological properties^11^. Although SWI has been used to evaluate the microhemorrhage, neovascularization, and preoperative grading of gliomas^12^, only a few previous studies have attempted to analyze the biological characteristics of gliomas using SWI. In this study, SWI images of gliomas were collected to analyze the correlations between imaging features and key molecular markers such as *IDH1*, *MGMT*, and 1p19q in order to reveal the characteristics of molecular markers associated with glioma microbleeds, which can provide a scientific basis for guiding individualized surgery in the future.

## Results

### Patient characteristics

The clinical, molecular pathological, radiological, and SWI data of 122 patients with gliomas were collected. The results are shown in Tables 1 and 2. Patients were divided into three groups according to pathological characteristics: WHO II, 60 cases (diffuse astrocytoma: 34 cases; oligodendroglioma: 25 cases; pleomorphic xanthoastrocytoma: one case); WHO III, 27 cases (anaplastic astrocytoma: 23 cases; anaplastic oligodendroglioma: four cases); and WHO IV, 35 cases. The patients were also classified by ITSS grade, with the following results: ITSS grade 0, nine cases; ITSS grade 1, 56 cases; ITSS grade 2, 13 cases; and ITSS grade 3, 44 cases. Among these patients, 85 patients underwent *IDH1* testing and 61 of them showed *IDH1* mutations; 101 patients underwent *MGMT* examinations and 82 showed *MGMT* methylation; and 88 patients underwent 1p19q testing and 30 showed a combined 1p19q deletion. In addition, ITSS grade was not related to sex or age. The agreement in the assessment of ITSS grades between the two neurosurgeons was 97.5%. In the 2.5% (3/122) of cases where the two raters disagreed, a third senior neurosurgeon made the decision.

**Table 1:** Clinical characteristics of the patients.

Characteristics	ITSS grade					
	ITSS 0	ITSS 1	ITSS 2	ITSS 3	Total	P-value^a^
Age (y)						0.346
Median (range)	44 (25–61)	42 (21–58)	42 (18–65)	45 (16–70)	43 (16–70)	
40 or older/40 or younger	6/3	31/25	8/5	30/14	75/47	
Sex						0.799
Male/female	4/5	33/23	7/6	23/21	67/55	
WHO grade						<0.001
LGG (WHO II)	7	43	8	2	60	
HGG (WHO III-IV)	2	13	5	42	62	
*IDH1* status						<0.001
Mutant	5	37	9	10	61	
Wild-type	0	5	1	18	24	
*MGMT* status						<0.001
Methylation	9	43	10	19	81	
Non-methylation	0	3	1	16	20	
1p19q status						0.186
Codeletion	2	19	5	4	30	
Non-codeletion	7	24	7	20	58	

^a^Results of Mann Whitney test. LGG = low-grade gliomas, HGG = high-grade gliomas.

**Table 2:** Expression status of molecular markers in histological types of gliomas.

Histological types	*IDH1* status		*MGMT* status		1p19q status	
	Mutant	Wild-type	Methylation	Non-methylation	Codeletion	Non-codeletion
**WHO II**						
diffuse astrocytoma	19	4	27	0	5	19
oligodendroglioma	23	0	25	1	18	9
pleomorphic xanthoastrocytoma	1	0	1	0	0	0
**WHO III**						
anaplastic astrocytoma	7	6	9	5	4	10
anaplastic oligodendroglioma	2	0	4	1	3	3
**WHO IV**						
glioblastoma	9	14	16	12	0	17
Total	61	24	82	19	30	58

### Association Between ITSS Grade and Histological Grade and *IDH1*, *MGMT*, and 1p19q Status

**Figure 1:**
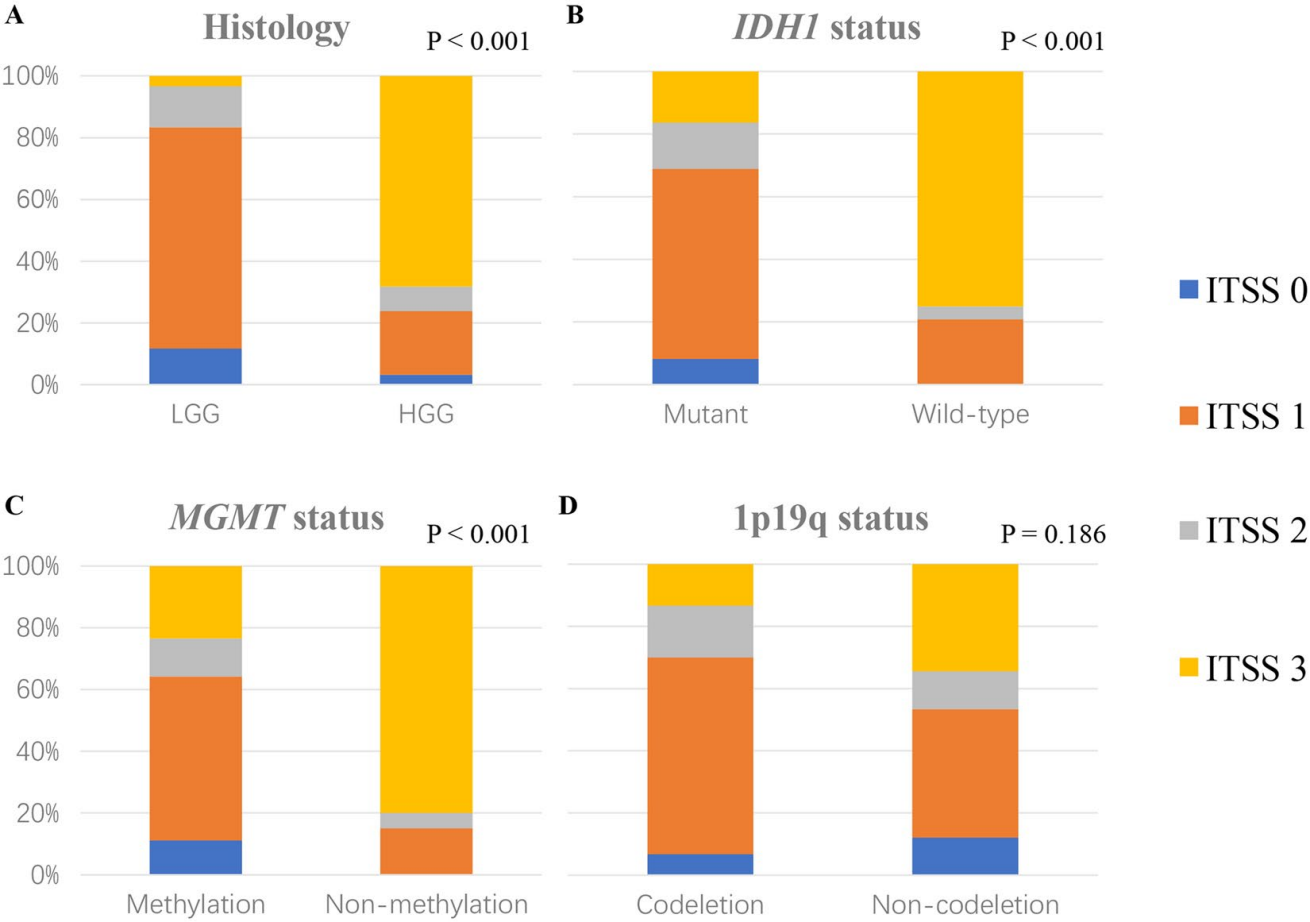
Histograms (using Mann Whitney test analyses) showing the associations between ITSS grade and pathological grade as well as *IDH1*, *MGMT*, and 1p19q status. (**A**) ITSS grades of high-grade gliomas were significantly higher than those of low-grade gliomas (*p* < 0.001); (**B**) ITSS grades of *IDH1* mutant gliomas were significantly lower than those of *IDH1* Wild-type gliomas (*p* < 0.001); (**C**) ITSS grades of *MGMT*-methylated gliomas were significantly lower than those of *MGMT*-unmethylated gliomas (*p* < 0.001); (**D**) There was no significant difference between ITSS grade and the expression status of 1p19q (*p* = 0.186).

Our study shows that the ITSS grades of HGG were significantly higher than those of LGG (*p* < 0.001). Similarly, the ITSS grades of *IDH1*-mutated gliomas were much lower than those of Wild-type gliomas (*p* < 0.001), and the ITSS grades of *MGMT*-methylated gliomas were much lower than those of *MGMT*-unmethylated gliomas (*p* < 0.001). However, there were no significant differences in 1p19q deletion status in relation to ITSS grade (*p* = 0.19) (Fig. 1).

The preoperative SWI images and expression status of molecular markers were analyzed, and the results are summarized in Table 1. Among the patients with ITSS grade 3, 28 underwent *IDH1* testing, and 10 (36%) of these patients showed *IDH1* mutations; 35 underwent *MGMT* testing and 19 (54%) showed *MGMT* methylation; and 24 underwent 1p19q examinations and four (17%) showed 1p19q codeletions. However, among patients with ITSS grade 1, 42 underwent *IDH1* testing and 37 (88%) of them showed *IDH1* mutations; 46 underwent *MGMT* testing and 43 (93%) showed *MGMT* methylation; and 43 underwent 1p19q examinations and 19 (44%) showed 1p19q codeletions. More interestingly, all patients diagnosed with ITSS grade 0 gliomas showed *IDH1* mutations and *MGMT* promoter methylation.

We analyzed the association between ITSS grades and *IDH1*, *MGMT*, and 1p19q status in the LGG group and the HGG group, respectively (Table 3). In the HGG group, ITSS grades of *IDH1*-mutated gliomas were significantly lower than those of Wild-type gliomas (*p* < 0.01). In addition, ITSS grades of *MGMT*-methylated gliomas were lower than those of *MGMT*-unmethylated gliomas (*p* = 0.041).

**Table 3:** Expression status of molecular markers in LGG and HGG.

Characteristics	ITSS grade					
	ITSS 0	ITSS 1	ITSS 2	ITSS 3	Total	P-value^a^
**LGG**						
*IDH1* status						>0.99
Mutant	3	32	6	2	43	
Wild-type	0	3	1	0	4	
*MGMT* status						>0.99
Methylation	7	37	7	2	53	
Non-methylation	0	1	0	0	1	
1p19q status						0.206
Codeletion	2	15	5	1	23	
Non-codeletion	5	19	3	1	28	
**HGG**						
*IDH1* status						<0.01
Mutant	2	5	3	8	18	
Wild-type	0	2	0	18	20	
*MGMT* status						0.041
Methylation	2	6	4	17	29	
Non-methylation	0	2	0	16	18	
1p19q status						0.273
Codeletion	0	4	0	3	7	
Non-codeletion	2	5	4	19	30	

^a^Results of Mann Whitney test. LGG = low-grade gliomas, HGG = high-grade gliomas.

### ITSS grade predicts pathological grade and molecular biomarkers

**Figure 2:**
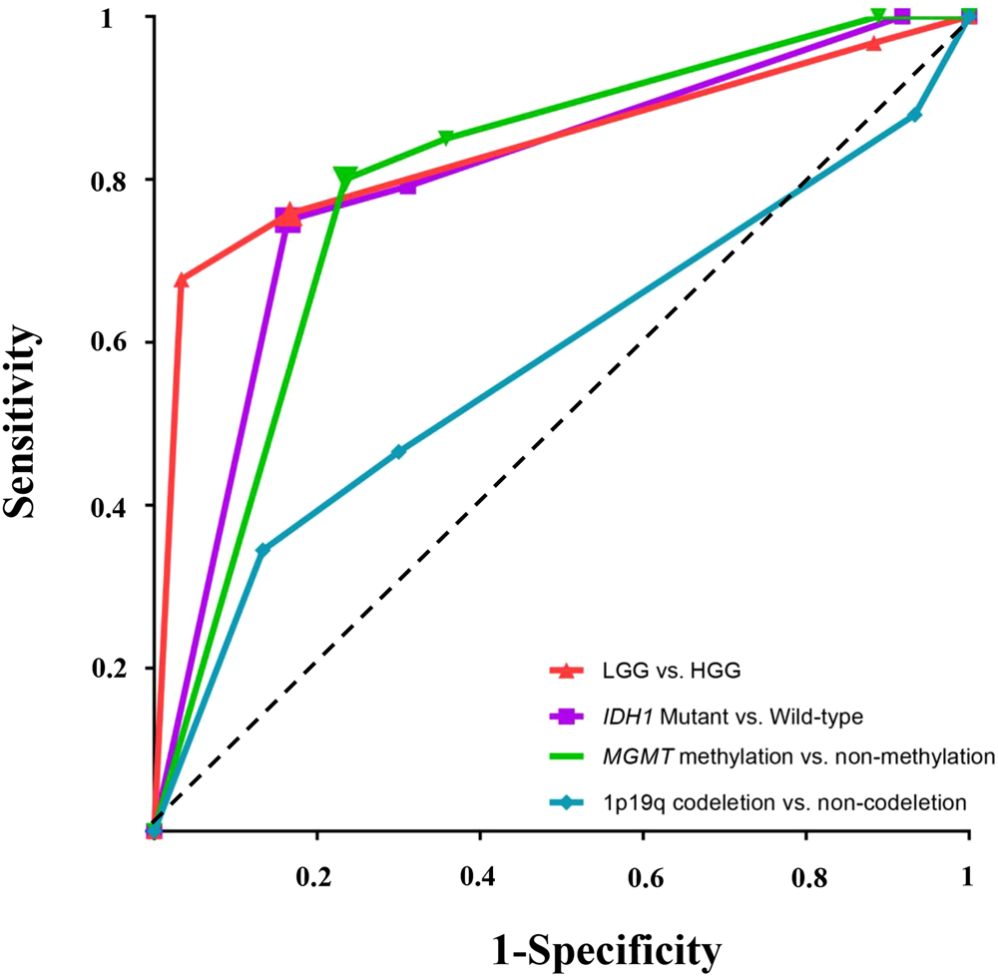
ROC curves used to assess the role of ITSS grades in predicting pathological grades and molecular biomarkers. The sensitivity results are plotted against 1-specificity to compare ITSS grades with pathological grades and molecular biomarkers. The dotted line is the reference line. The bold points represent the optimal cutoff point. ROC of low-grade gliomas (LGG) *vs*. high-grade gliomas (HGG) (1.5 was the best cutoff value with 75.8% sensitivity, 83.3% specificity, and AUC = 0.84); ROC of *IDH1* mutant *vs*. Wild-type (2.5 was the best cutoff value with 75.0% sensitivity, 83.6% specificity, and AUC = 0.80); ROC of *MGMT* methylation vs. non-methylation (2.5 was the best cutoff value with 80.0% sensitivity, 76.5% specificity, and AUC = 0.79); ROC of 1p19q codeletion *vs*. non-codeletion (AUC = 0.58).

We analyzed the role of the glioma ITSS grade in predicting pathological grade and the expression status of *IDH1*, *MGMT*, and 1p19q. The ITSS grade showed a significant effect in predicting pathological grade and the expression status of *IDH1* and *MGMT*. The receiver operating characteristic (ROC) curve analysis indicated that 1.5 was the best cutoff value (75.8% sensitivity; 83.3% specificity; AUC = 0.84) to predict pathological grade, 2.5 was the optimal cutoff value (75.0% sensitivity; 83.6% specificity; AUC = 0.80) to predict *IDH1* status; and 2.5 was the best cutoff value (80.0% sensitivity; 76.5% specificity; AUC = 0.79) to predict *MGMT* status. However, the ITSS grade showed no significant effect in predicting the expression status of 1p19q (AUC* = *0.58) (Fig. 2).

## Discussion

The glioma microenvironment reflects biological characteristics of tumor lesions. SWI sequences are very sensitive in the detection of blood and vascular structures and can show microhemorrhages as well as neoangiogenesis, which are not visible on conventional MRI sequences. We assessed the SWI signals of patients with gliomas to determine ITSS and pathological grades as well as expression levels of molecular markers, to assess correlations between ITSS grades and pathological grades, *IDH1* mutation status, *MGMT* promoter methylation status, and 1p19q deletion status.

In previous studies, SWI and SWI-based ITSS grading were widely used in the preoperative diagnosis of brain tumors. In cases of gliomas, this approach plays an especially important role in tumor grading, assessments of calcification, hemorrhage and neovascularization, differential diagnoses between gliomas and other intracranial lesions, and post-treatment monitoring. SWI has significant advantages in the identification of gliomas, lymphomas, metastases, meningiomas, and brain abscesses^13^, and can also be used to non-invasively identify areas of tumor invasion^14^. Additionally, it can help determine vascular structural changes in patients with glioblastomas and monitor the effects of anti-angiogenic therapy, cytotoxic chemotherapy, and radiation therapy *in vivo*, serving as an effective tool for neuroradiological follow-up^15^. Moreover, in terms of the preoperative grading of gliomas, SWI, on the basis of conventional imaging techniques, could potentially be used to complement other techniques (i.e., inclusion in radiomics models, combination with perfusion parameters). Statistical comparisons show significant differences in hemorrhage in small blood vessels and microvessels between gliomas of different grades, with higher tumor grades indicating greater presence of small blood vessels and hemorrhage in the tumor. Therefore, the higher the grade of a glioma, the higher its ITSS grade^16^. The classification of gliomas is not only based on histopathology but also on the molecular characteristics of the tumor, which are crucial for the treatment and prognosis of glioma^17^. This study used a standard SWI grading method to identify correlations between ITSS imaging features and tumor as well as molecular pathology, and confirmed the findings of previous studies that showed that SWI plays an important role in predicting the status of molecular glioma markers.

The latest research findings show that some specific molecular markers are closely related to the origin and malignant progression of the glioma^3^. Among the gene markers of gliomas, *IDH* gene mutations and *MGMT* methylation are the most critical, and these mutations have an important influence on the prognosis of gliomas. Previous studies have confirmed that *IDH1* mutations lead to the activation of the hypoxia-inducible factor (HIF)-1α pathway^18^, which may be one of the carcinogenic mechanisms. The activity of HIF-1α plays a central role in maintaining energy metabolism, tumor angiogenesis, and promoting tumor cell proliferation and metastasis. Polívka *et al*.^19^ found that *IDH* mutations were associated with lower expression of vascular endothelial growth factor (VEGF). Another recent study found that platelet derived growth factor receptor alpha (PDGFRA) is enriched in *MGMT*-methylated gliomas^20^, which plays a role in platelet activation, secretion of agonists from platelet granules, and in thrombin-induced platelet aggregation. The process of angiogenesis is turned on by the presence of hypoxia that results in upregulation of HIF1α, which subsequently leads to the upregulation of vascular permeability factors such as VEGF^21^. PDGF was also identified to play an important role in tumor angiogenesis. These findings suggest that *IDH1* mutations and *MGMT* methylation are associated with tumor angiogenesis. ITSS reflects microhemorrhage in gliomas, as described previously. Our study found that *IDH1* mutations and *MGMT* methylation are related to ITSS grading, providing new evidence for the relation between imaging findings and molecular genetic mechanisms associated with angiogenesis resulting from *IDH1* mutations and *MGMT* methylation.

With advancements in imaging technology, simple structural images have evolved to multi-parametric functional images, which can non-invasively and dynamically reflect the microenvironment, blood supply, and metabolism of tissues (cells)^22^. Non-invasive MRI can help provide a more comprehensive perspective on tumor molecular and pathological profiles by providing complementary information on spatial heterogeneity and temporal changes compared to molecular and conventional pathology alone, which often relies on focal surgical sampling. In addition, the non-invasive nature of MRI can facilitate periodic monitoring of the malignant progression of tumors, which has much significance in predicting the prognosis of patients and evaluating therapeutic effects. Research studies have successfully combined imaging with genomics to evaluate clinical prognoses in cases of lung cancer, head and neck cancer, and gliomas^23,24^. For example, a recent study found that the apparent diffusion coefficient (ADC) and relative cerebral blood flow (rCBF) are promising imaging biomarkers to predict *MGMT* methylation in patients with glioma^25^. Multimodal MRI was also performed for molecular typing in gliomas, and proved to be effective in predicting the expression of *IDH1* in glioma^26^. Although there are numerous studies on SWI, most have focused on glioma grading and few have combined SWI with genetic markers. However, to the best of our knowledge, this is the first study that combines SWI and glioma biomarkers. The advantage of SWI over advanced functional imaging lies in better detection of micro-bleeding and angiogenesis. We found that these imaging characteristics are related to the status of tumor molecular markers. This provides new ideas for the non-invasive prediction of the molecular genetics of gliomas, and also an important basis for preoperative personalized surgical treatment based on molecular pathology.

Some limitations of our study should also be considered. SWI imaging features are related to the amount of internal bleeding in the tumor. A small amount of bleeding may exaggerate the low signal area, which can obscure other structures in the lesion; moreover, the significantly low signal area caused by extensive hemorrhage may conceal the blood vessels inside the tumor and hinder the correct assessment of the blood vessels. Therefore, it is not easy to use SWI to perform quantitative analyses, but, currently, it is often necessary to use ITSS grading. ITSS scores are generally a reliable and stable basis for classifying SWI findings of gliomas. In our study, the assessment of ITSS grades was highly consistent between the two neurosurgeons. Only in 2.5% of cases, disagreements between the first two neurosurgeons occurred, probably caused by a judgment error of one rater. Besides, improvements in scanning parameters can enhance SWI to achieve an even more detailed depiction of venules and hemorrhages in gliomas, thereby providing more comprehensive information regarding the internal structures of the tumor. Despite these limitations, SWI is an excellent sequence for observing the internal components of tumors. Future research should combine SWI features with genetic features to explore the genetic mechanisms associated with intratumoral hemorrhage.

## Methods

### Patients

The study protocol was approved by the ethics committee of Beijing Tiantan Hospital and all procedures were in accordance with the institutional guidelines and regulations. The findings for 122 adult patients who were diagnosed with gliomas and underwent surgical therapy at Beijing Tiantan Hospital from December 2013 to August 2017 were retrospectively reviewed. All study participants aged 18 or more provided signed informed consent, and for those under the age of 18, the parents provided informed consent according to the approved guidelines. Cases were included when they met the following criteria: (i) age 16 years or older; (ii) no previous diagnosis of any brain tumor, no history of brain biopsy or brain surgery, and no history of brain radiotherapy and chemotherapy; (iii) preoperative multimodal MRI data available (including T1-weighted, T2-weighted, postcontrast T1-weighted, and SWI data); (iv) pathologically confirmed glioma based on the 2016 classification of tumors of the central nervous system; and (v) molecular markers assessed for tumor samples. The histopathological diagnosis was evaluated and confirmed by two independent senior neuropathologists.

### Image acquisition

MR imaging was performed using a Siemens Trio 3.0 T scanner (Siemens Healthcare). It typically included axial T1-weighted (repetition time [TR], 2100 ms; echo time [TE], 2.5 ms; flip angle [FA], 7°) images with a field of view (FOV) and matrix size of 256 × 256 mm^2^ and acquisition voxel size of 1.0 × 1.0 × 1.0 mm^3^; T2-weighted images (TR, 5500 ms; TE, 120 ms) with an FOV and matrix size of 240 × 240 mm^2^, and acquisition voxel size of 1.0 × 1.0 × 5.0 mm^3^; gadolinium-DTPA (Gd-DTPA) injection-enhanced (0.1 mmol/kg) axial T1-weighted images (TR, 450 ms; TE, 15 ms; section thickness, 5 mm) with an FOV and matrix size of 240 × 240 mm^2^, and SWI images (TR, 36 ms; TE, 51 ms; FA, 15°; FOV, 23 × 19 cm; slice thickness, 0.6 mm; slice gap, 0 mm; number of slices, 180; acquisition time, 278 s;) with an acquisition voxel size of 0.8 × 0.7 × 1.2 mm^3^ obtained using a 3-dimensional, high-spatial resolution, fully velocity-compensated gradient echo sequence. Postcontrast images were obtained immediately after injection of the contrast agent. The radiologic parameters in preoperative scans of all patients were maintained consistently.

### Identification of imaging features

**Figure 3:**
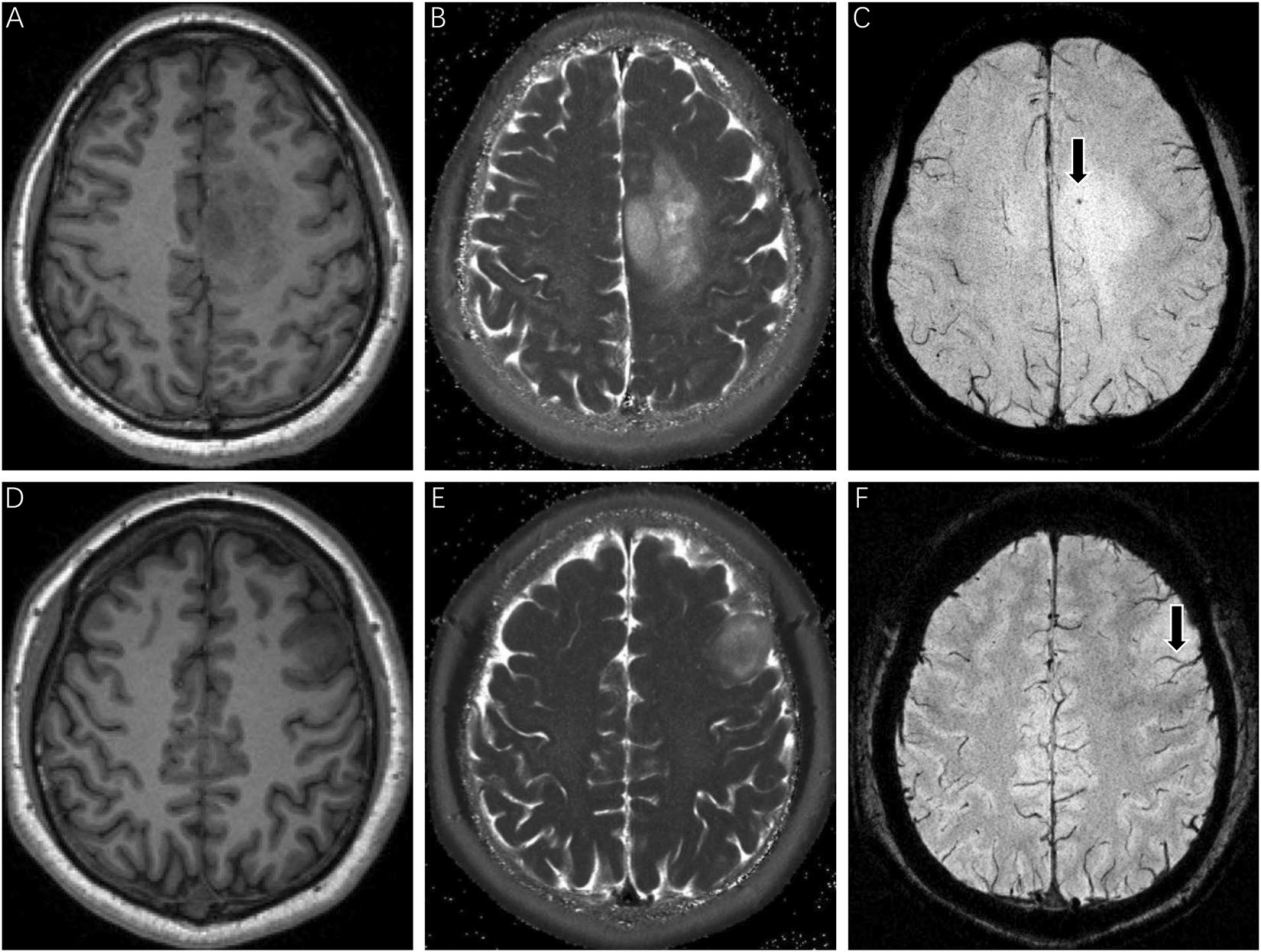
SWI imaging features of gliomas. SWI imaging (**C,F**) shows significant advantages in identifying microbleeds and microvessels in comparison with T1-weighted (**A,D**) and T2-weighted (**B,E**) imaging (as indicated by the arrows).

**Figure 4:**
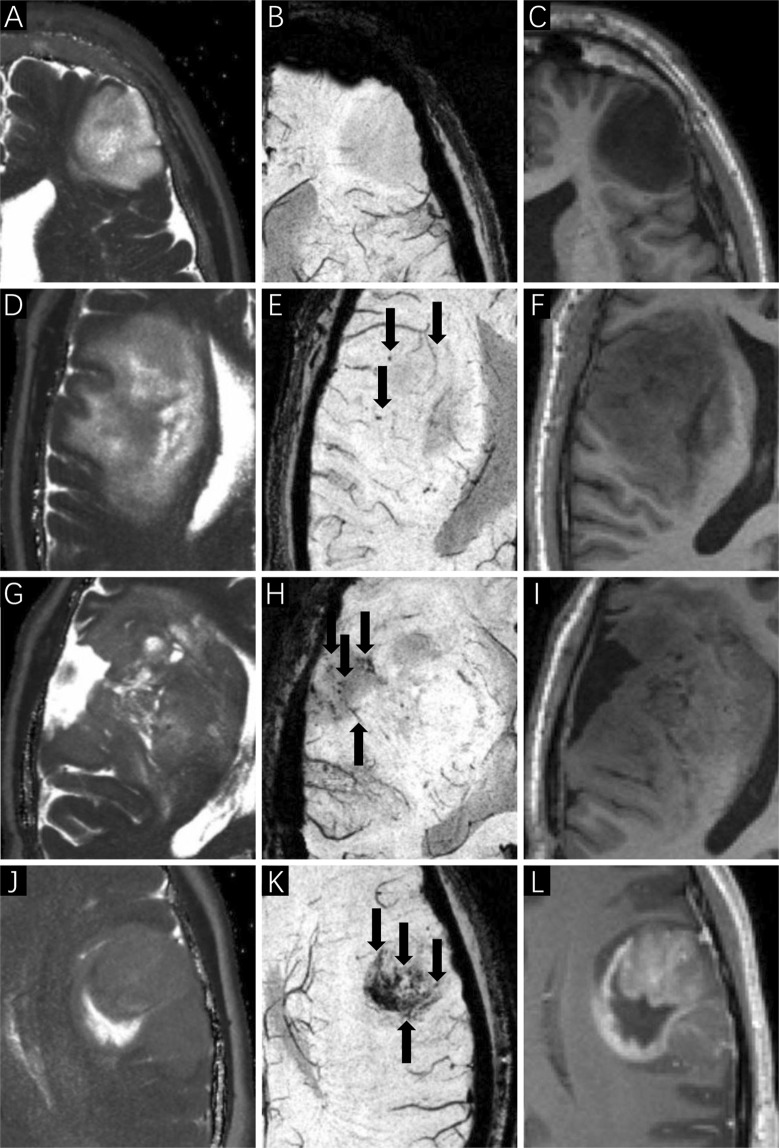
ITSS manifestation in different grades of gliomas (as indicated by the arrows). (**A–C**) Astrocytoma WHO II, ITSS 0; (**D–F**) oligodendroglioma WHO II, ITSS 1; (**G**–**I**). oligodendroglioma WHO II-III, ITSS 2; (**J–L**) glioblastoma WHO IV, ITSS 3.

**Figure 5:**
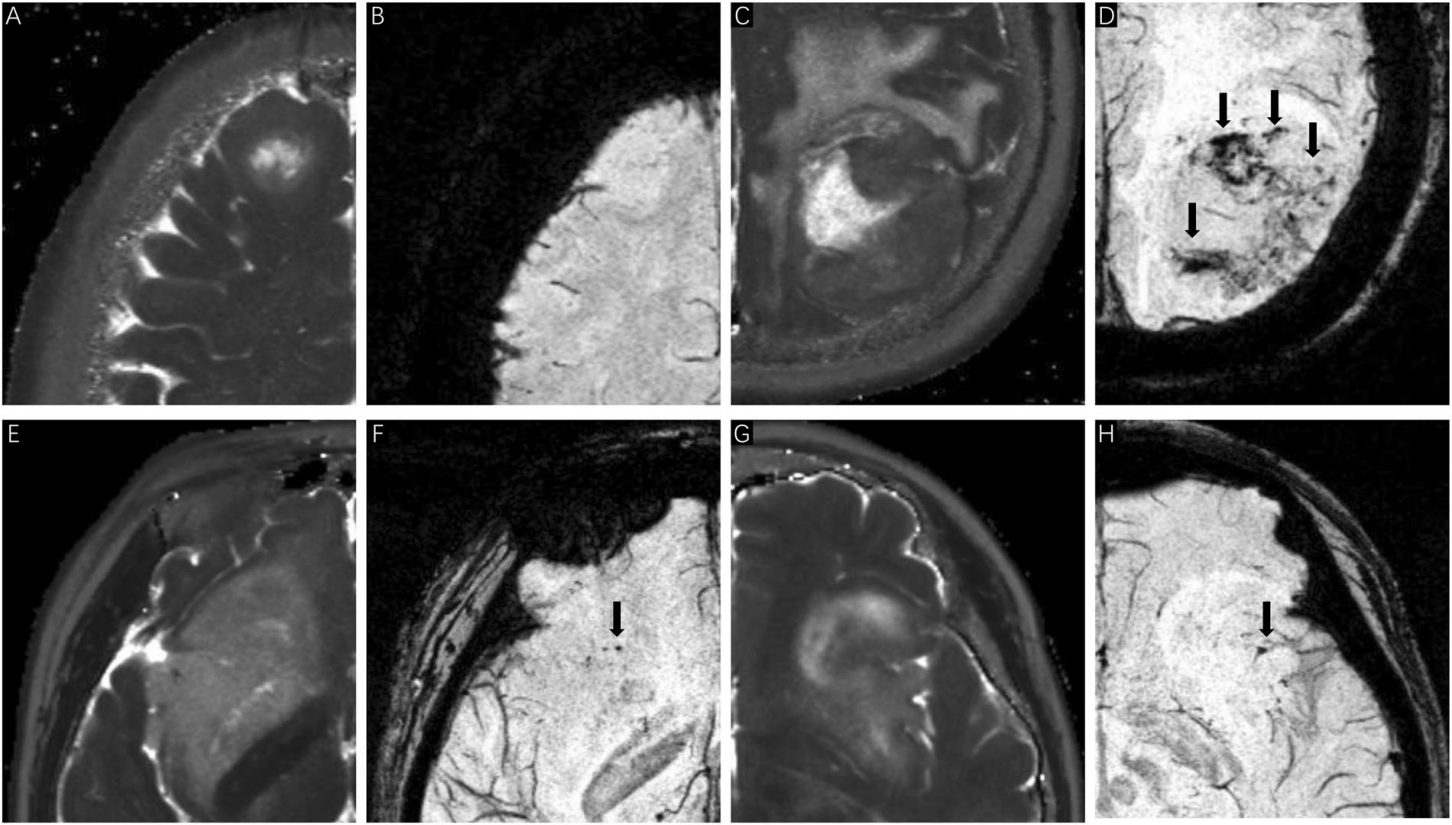
ITSS manifestation in relation to the expression status of different molecular markers (as indicated by the arrows). (**A,B**) Glioblastoma WHO IV, *IDH1* mutation, *MGMT* methylation, ITSS 1; (**C,D**) Glioblastoma WHO IV, *IDH1* Wild-type, unmethylated *MGMT*, ITSS 3. (**E**,**F**) Astrocytoma WHO II, 1p19q codeletion, ITSS 1; (**G**,**H**) Astrocytoma WHO II, 1p19q non-codeletion, ITSS 1.

The ITSS grades on SWI images were assessed by two experienced neurosurgeons who were blinded to the patients’ clinical information. A third senior neurosurgeon re-examined the images and determined which should be used if the ITSS grades identified by the first two neurosurgeons were inconsistent. ITSS^27^ is defined as linear or dot-like areas of low signal gathering or scattering in the tumors, which can be clearly observed on SWI in comparison with conventional MRI (Fig. 3). Three types of ITSS were identified in accordance with the morphological features: collection of dot-like structures, collection of fine linear structures, and the mixed structure described above. Therefore, the ITSS grading scheme was based on the number of linear or dot-link structures in the maximum cross-section of tumors on SWI, and the scheme was as follows: Grade 0: no ITSS; Grade 1: 1–5 dot-like or fine linear ITSS; Grade 2: 6–10 dot-like or fine linear ITSS; and Grade 3: 11 dot-like or fine linear ITSSs (Figs. 4 and 5).

### Molecular pathology detection

*IDH1* mutation proteins were detected by immunohistochemistry, and proteins showing negative expression were used to determine the *IDH1* mutation status by using DNA pyrosequencing^28^, First, complete genomic DNA was extracted from tumor tissue samples using a QIAamp DNA Mini Kit (QIAGEN, Hilden, Germany). Second, the genomic region spanning the Wild-type R132 of *IDH1* was amplified with the primers 5′-GCTTGTGAGTGGATGGGTAAAAC-3′ and 5′-biotin-TTGCCAACATGACTTACTTGATC-3′. Then, the *MGMT* methylation status was determined by pyrosequencing using a 5′- GGATATGTTGGGATAGT-3′ primer aimed at the *MGMT* promoter region. A total of seven methylation sites were detected, and a methylation percentage greater than 10% was considered to indicate hypermethylation. Finally, fluorescence *in situ* hybridization was used to identify 1p19q deletion status.

### Statistical analysis

We used the Mann Whitney test in GraphPad Prism 7 for P-values and drew histograms to compare the ITSS grades of gliomas with pathological grades, *IDHI* mutation status, *MGMT* methylation status, and combined 1p19q deletion status. In addition, AUC values were calculated from the ROC curve that was obtained with GraphPad Prism 7 to analyze the role of ITSS grading in predicting pathological grades and the expression status of *IDH1*, *MGMT*, and 1p19q.
